# Initial experience of single-incision plus one port total laparoscopic pancreaticoduodenectomy

**DOI:** 10.1186/s12893-023-02107-2

**Published:** 2023-08-07

**Authors:** He Cai, Man Zhang, Xin Wang, Yunqiang Cai, Bing Peng

**Affiliations:** 1 Department of General Surgery, Division of Pancreatic Surgery, West China Hospital, Sichuan University, No. 37, Guo Xue Xiang, Chengdu, 610041 Sichuan China; 2 Department of Minimal Invasive Surgery, Shangjin Nanfu Hosptial, Chengdu, China

**Keywords:** Pancreatoduodenectomy, Laparoscopy, Single-incision plus one port, Pain score, Cosmetic result

## Abstract

**Background:**

The use of single-incision plus one-port laparoscopic pancreaticoduodenectomy (SILPD + 1) has been never reported, and its safety and efficacy remain unknown. This study aimed to evaluate the short-term outcomes of SILPD + 1 compared with those of conventional laparoscopic pancreaticoduodenectomy (CLPD).

**Method:**

Fifty-seven cases of laparoscopic pancreaticoduodenectomy (LPD) were performed between November 2021, and March 2022. Among them, 10 cases of LPD were performed using a single-incision plus one-port device. Based on the same inclusion and exclusion criteria, 47 cases of LPD performed using traditional 5-trocar were included as a control group. The patient’s demographic characteristics, intraoperative, and postoperative variables were prospectively collected and retrospectively analyzed.

**Results:**

Three men and seven women were included in the SILPD + 1 group. All baseline parameters of both groups were comparable, except for age. Patients were younger in the SILPD + 1 group (47.2 ± 18.3 years vs. 60.6 ± 11.7 years, *P* = 0.05) than that in the CLPD group. Compared with the CLPD group, median operation time (222.5 (208.8–245.0) vs. 305.0 (256.0–37.0) min, *P* < 0.001) was shorter, median postoperative VAS scores on days 1–3 were lower, and median cosmetic score (21.0 (19.0–23.5) vs. 17.0 (16.0–20.0), *P* = 0.026) was higher one month after the surgery in the SILPD + 1 group. The estimated blood loss, conversion rate, blood-transfusion rate, exhaust time, time of drainage tube removal, postoperative hospital stays, and perioperative complications were comparable between the two groups.

**Conclusion:**

In a high-volume LPD center, SILPD + 1 is safe and feasible for well-selected patients without increasing the operation time and complications. It even has the advantages of reduced postoperative pain and improved cosmetic results.

**Supplementary Information:**

The online version contains supplementary material available at 10.1186/s12893-023-02107-2.

## Introduction

In 1992, Gagner and Pomp reported the world's first case of laparoscopic pancreaticoduodenectomy (LPD) [1]. Nowadays LPD is now approved as safe and feasible in high-volume centers and may be associated with a shorter time to functional recovery and shorter hospital stay than open pancreaticoduodenectomy (OPD) [2, 3]. However, combining a challenging resection and multiple anastomoses remains a technical difficulty. Single-incision laparoscopic surgery (SILS) has been widely used in gynecological and thoracic surgery with many benefits, including a reduced risk of trocar-related complications, reduced postoperative pain, and improved convalescence and cosmetic results [4, 5]. However, only a few single-incision LPD (SILPD) cases have been described [6]. From February 2020 to December 2020, we finished 13 SILPDs and found them feasible with several potential advantages mentioned above [7]. However, SILPD is much more challenging owing to a limited range of motion, instrument crowding, and collision, difficulty in bleeding control, and longer operative time [7]. Single-incision plus one-port surgery has been reported in distal pancreatectomy and rectosigmoid cancer as possibly safer and more feasible without losing those benefits compare with SILS [8, 9]. However, the safety, efficacy, and advantages of single-incision plus one-port LPD (SILPD + 1) have never been reported. In this article, we decided to evaluate the short-term outcomes of SILPD + 1 compared with those of conventional LPD (CLPD).

## Methods

### Study design and patients

From November 2021 to March 2022, 57 cases of LPD were performed by a single surgical team in the Department of Pancreatic Surgery, West China Hospital, Sichuan University. Data were prospectively recorded in the database. Among these patients, 10 accepted SILPD + 1 (SILPD + 1 group), and 47 accepted CLPD (CLPD group). The inclusion criteria of SILPD + 1 and CLPD were almost the same, except that we tended to choose lower body mass index (BMI) and younger patients for SILPD + 1 just as SILPD [7]. The final decision on the surgical approach (SILPD + 1 or CLPD) was made by the surgeon and patient. We decided to perform only OPD for patients with suspected artery involvement (4 patients) or those who rejected to accept laparoscopic surgery (2 patients). For the remaining patients, we adopted the policy of attempting laparoscopic surgery, and 10 patients were actively converted to laparotomy by the surgeon in the exploration phase. Thus, these 16 patients were excluded from this study. Data in terms of demographic characteristics (surgical approach, age, sex, BMI, American Society of Anesthesiology (ASA) score, hemoglobin, total bilirubin, tumor size, pathological diagnosis, pancreatic duct diameter, and gland texture), intraoperative variables (venous resection, estimated blood loss, and transfusion) and postoperative variables (postoperative hospital stay, visual analog scale (VAS), exhaust time, time of drainage tube remove, and complications) were collected. Follow-up data were collected one month after the surgery. Informed consent was obtained from all patients, and the study was approved by the Ethics Committee of Sichuan University (approval number 2021(1040)).

### Perioperative management

All patients underwent enhanced computed tomography preoperatively to confirm the diagnosis and evaluate the extent of the tumor. Other routine examinations were performed, such as blood tests, electrocardiograms, and chest X-rays. Percutaneous transhepatic choledochus drainage was selectively performed for patients with severe jaundice to facilitate biliary drainage preoperatively. A nasogastric tube (NGT) was used during surgery and removed 1–2 days after surgery, and oral intake was advanced as tolerated [10]. Serum and drainage amylases were routinely assessed on postoperative days (PODs) 1, 3, 5, and 7. Computed thoracic and abdominal cavities tomography was performed on POD 4 or 5. The abdominal drainage was removed for patients with drainage amylase lower than 3000 U/L when computed tomography demonstrated no abnormal findings. Patients were discharged when oral intake and moderate activity were tolerated without any abnormal postoperative complications or laboratory findings [7].

### Operation process (Video 1)

**Fig. 1:**
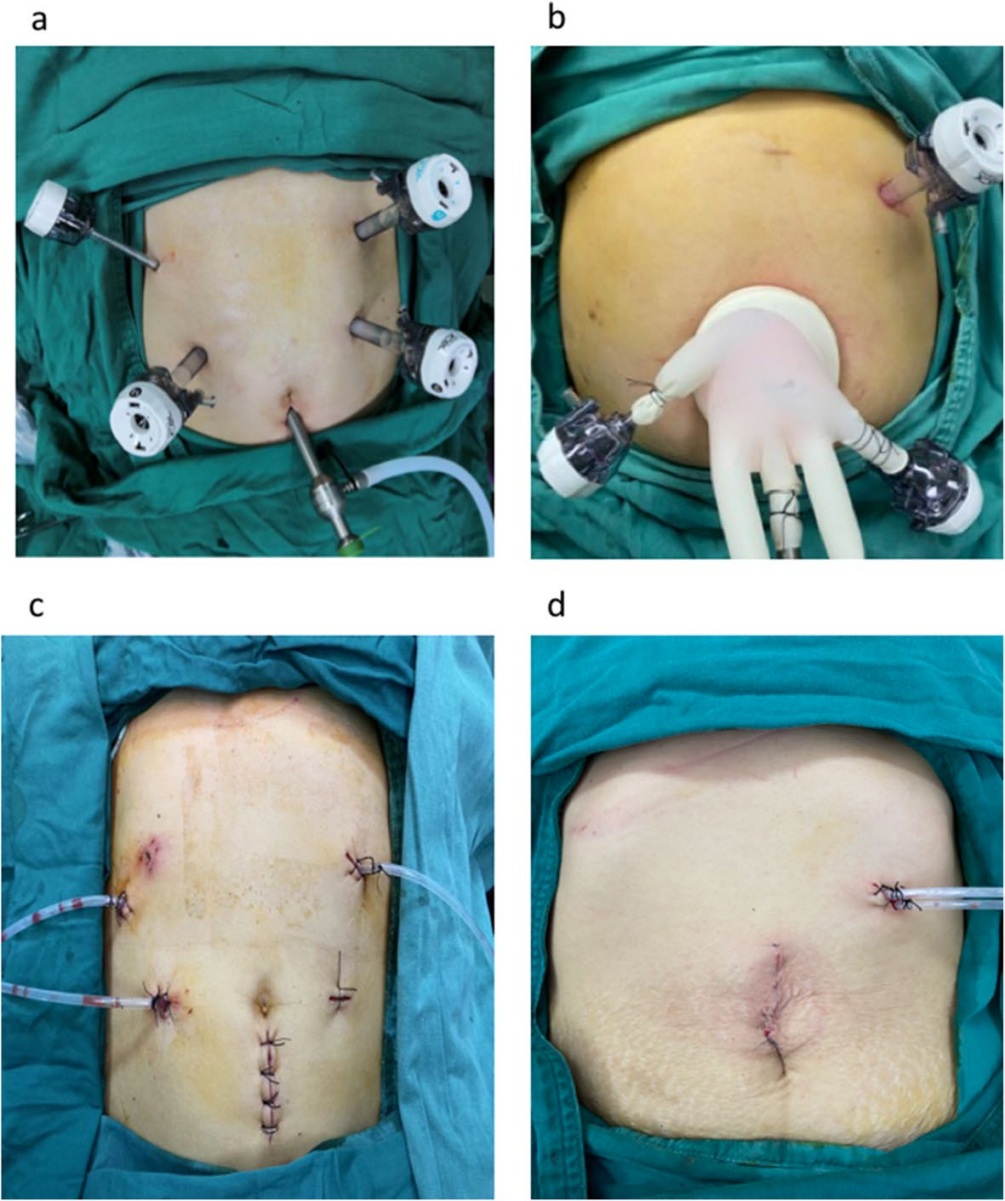
**a** Trocar distributions in the CLPD group, five trocars (three 12 mm trocars, a 10 mm trocar, and a 5 mm trocar) were placed in a “V”-shape. **b** Trocars distributions in the SILPD + 1 group, a 4 cm incision was made in the umbilical midline. Three trocars (a 12 mm, a 10 mm, and a 5 mm trocar) were inserted through the glove fingers. **c** abdominal cavity drainage in the CLPD group; **d** abdominal cavity drainage in the SILPS + 1 group. CLPD, conventional laparoscopic pancreaticoduodenectomy, SILPD + 1 single-incision plus one-port laparoscopic pancreaticoduodenectomy

**Fig. 2:**
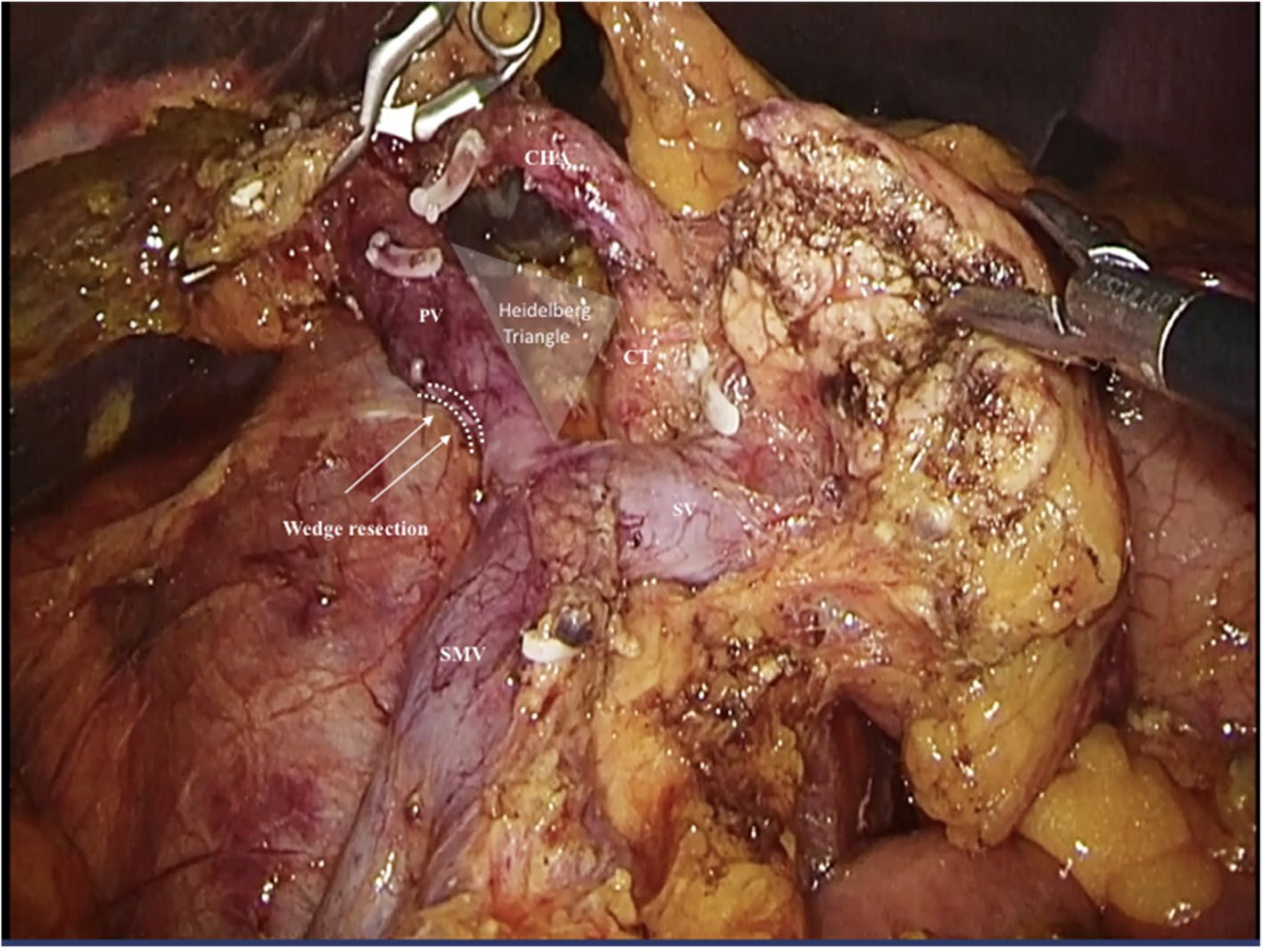
Surgical field of SILPD + 1 with PV wedge resection. PV, portal vein; SMV, superior mesenteric vein; CHA, common hepatic artery; SV, splenic vein; CT, coeliac trunk

Under general anesthesia, the patient was placed in the supine position with legs apart. A 10 mm trocar was placed for the telescope. For the CLPD group, five trocars (three 12 mm trocars, a 10 mm trocar, and a 5 mm trocar) were placed in a “V”-shape (Fig. 1a). For the SILPD + 1 group, a 4 cm incision was made in the umbilical midline. Three trocars (a 12 mm, a 10 mm, and a 5 mm trocar) were inserted through the glove fingers. Then, a homemade multichannel device comprising a soft-tissue retractor with a surgical glove was inserted. An additional 12 mm trocar was placed slightly lateral to the left midclavicular line at the level of 1 cm below the costal edge (Fig. 1b). For the CLPD group, the CO_2_ pneumoperitoneum was established at 12–13 mmHg. For the SILPD + 1 group, the CO_2_ pneumoperitoneum was established at 8–10 mmHg. The surgical technique has been described previously [11, 12]. In a typical procedure, the gastrocolic ligament was opened after exploration, and the hepatic flexure of the colon was fully taken down. Next, an extended Kocher maneuver was performed. Then, the duodenum, gastroduodenal artery, common hepatic duct, pancreatic neck proximal jejunum, and uncinate process of the pancreas were dissected (Fig. 2). Finally, pancreatojejunostomy was performed in Bing’s duct-to-mucosa manner [12] which has been described previously. A hepaticojejunostomy was created as an end-to-side anastomosis. We used extracorporeal gastrojejunostomy for SILPD + 1 and intracorporeal gastrojejunostomy for CLPD. For the CLPD group, the specimen was removed via an enlarged trocar site, and the abdominal cavity was drained through other trocar sites (Fig. 1c). For the SILPD + 1 group, the specimen was removed via the incision, and the abdominal cavity was drained through the trocar site (Fig. 1d).

### Definition

This study used the International Study Group of Pancreatic Surgery’s definitions of pancreatic fistula and post pancreatectomy hemorrhage (PPH) [13, 14]. Follow-up data included the cosmetic score which was defined by Dunker [15]. Mortality was defined as death that occurred within 90 days of surgery. The length of postoperative hospital stay was calculated from the day of surgery up to, and including, the day of discharge. Postoperative complications were defined according to the Clavien – Dindo classification of surgical complications [16].

### Statistical analysis

Statistical analysis was performed with SPSS 24.0 software. Quantitative results are expressed as median (interquartile range) or median (range), whereas categorical data are expressed as numbers and percentages of cases. Categorical variables were assessed using the χ2 or Fisher exact test. Comparisons of continuous data were performed with the use of the Student t-test for normally distributed data; otherwise, the Man–Whitney U test was used. The rejection level for the null hypothesis was set at a *P* value of < 0.05.

## Results

The demographic characteristics and perioperative outcomes of patients who underwent SILPD + 1 are shown in Table 1. The patient population included three male and seven female patients with a mean age of 47.2 (range = 18.0–73.0) years and a median BMI of 21.0 (range = 19.8–27.1) kg/m^2^. The postoperative pathologic diagnosis included five cases of pancreatic ductal adenocarcinoma and one case each of duodenal papilla tumor, pancreatic neuroendocrine tumor, serous cystadenoma, pancreatic neuroendocrine tumor, and chronic pancreatitis. Two (20%) patients had portal-vein involvement with wedge resection. The median operative time was 222.5 (range 194.0–300.0) min. The median blood loss was 75.0 (range = 50–250) mL. One (10%) patient developed a pancreatic fistula (grade B). One (10%) patient had a major postoperative complication (Clavien–Dindo ≥ grade 3) requiring reoperation in laparoscope as afferent loop obstruction. However, no patient suffered postoperative bleeding, biliary leakage, and grade C pancreatic fistula. No 90-day mortality was found.

**Table 1:** Data of the patients who underwent single-incision plus one-port laparoscopic pancreaticoduodenectomy

Patients No	Sex	Age	BMI (Kg/m^2^)	Diagnosis	Tumor size (cm)	Operative time(min)	VR	No. of lymph node harvested	EBL (ml)	POHS (d)	Complications
1	Female	24	25.1	VPC	1.1	223	No	7	20	9	None
2	Male	65	27.1	PDCA	1.8	260	No	25	150	15	Biochemical leakage
3	Male	58	20.6	CP	2.0	220	No	NN	250	17	Biochemical leakage
4	Male	38	22.7	PDCA	2.2	300	Yes	22	200	38	Pancreatic fistula (Grade B), Chylous leakage
5	Female	55	20.4	PDCA	2.0	240	Yes	19	100	25	Diarrhea
6	Female	73	20.3	PDCA	4.0	205	No	18	50	10	None
7	Female	63	24.6	TDP	1.6	222	No	NN	30	10	None
8	Female	18	21.4	PNET	2.6	210	No	8	100	14	Biochemical leakage, Reoperation (Laparoscope)
9	Female	40	19.8	PDCA	4.0	230	No	20	26	8	Biochemical leakage, diarrhea
10	Female	38	20.0	SCA	5.2	194	No	NN	20	14	None

*BMI* Body mass index, *VR* Venous resection, *EBL* Estimated blood loss, *POHS* Postoperative hospital stay, *VPC* Vater ampulla carcinoma, *PDCA* Pancreatic ductal carcinoma, *CP* Chronic pancreatitis, *TDP* Tumor of duodenal papilla, *PNET* Pancreatic neuroendocrine tumor, *SCN* Serous cystadenoma, *NN* no need

The demographic characteristics of the two groups are compared in Table 2. The two groups did not statistically differ in sex, BMI, ASA score, laboratory examination, tumor size, pancreatic duct diameter, gland texture, and pathological diagnosis (all *P* > 0.05). Patients were younger in the SILPD + 1 group (47.2 ± 18.3 years vs. 60.6 ± 11.7 years, *P* = 0.05) than that in the CLPD group.

**Table 2:** The demographic characteristics of two groups

Variables	SILPD + 1 *n* = 10	CLPD *n* = 47	*P*
Age, mean (SD), y	47.2 (18.3)	60.6 (11.7)	0.050
Sex, (male/female), n	3/7	29/18	0.913
BMI, mean (SD), kg/m^2^	21.0 (2.6)	22.8 (3.6)	0.567
ASA score	_	_	1.000
II, n (%)	9 (90)	41 (87.2)	_
III, n (%)	1 (10)	6 (12.8)	_
History of abdominal surgery^a^, n (%)	1 (10.0)	8(17)	0.940
Preoperative biliary drainage, n (%)	1 (10.0)	7(14.9)	1.000
Hemoglobin, mean (SD), g/L	127.8 (15.4)	128.6 (24.8)	0.927
Albumin, mean (SD), g/L	39.7 (2.9)	40.3 (5.2)	0.627
TB, median (IQR), μmol/L	11.8 (8.0–43.8)	15.7 (10.5–86.0)	0.285
Tumor size, mean (SD), cm	2.7 (1.3)	2.5 (1.2)	0.711
Diameter of Pancreatic duct, median (IQR), mm	5 (2.8–6.3)	3 (2.5–5.0)	0.311
Gland texture (Firm/Soft), n	5/5	18/29	0.741
Diagnosis			0.819
Duodenum cancer, n (%)	0 (0)	4 (8.5)	_
PDAC, n (%)	5 (50.0)	17 (36.2)	_
CCBD, n (%)	0 (0)	3 (6.4)	_
CP, n (%)	1 (10.0)	2 (4.3)	_
PNET, n (%)	1 (10.0)	4 (8.5)	
Pancreatic cystic tumors^b^, n (%)	1 (10.0)	5 (10.6)	_
TDP, n (%)	1(10.0)	6 (12.8)	
Others^c^, n (%)	1 (0)	6 (12.8)	

*SILPD* + *1* Single-incision plus one-port laparoscopic pancreaticoduodenectomy, *CLPD* Conventional laparoscopic pancreaticoduodenectomy, *BMI* Body mass index, *ASA* Score American Society of Anesthesiologists classification score, *SD* Standard deviation, *IQR* Interquartile range, *TB *Total bilirubin, *PDCA* Pancreatic ductal carcinoma, *CCBD* Carcinoma of common bile duct, *CP* Chronic pancreatitis, *PNET* Pancreatic neuroendocrine tumor, *TDP* Tumor of duodenal papilla

^a^History of abdominal surgery includes cholecystectomy, appendectomy, biliary tract, and gastric surgery

^b^Pancreatic cystic tumors include serous cystadenoma, mucinous cystadenoma, and intrapancreatic mucinous papillary tumor

^c^Others include pancreatic head metastasis from clear cell renal cell carcinoma and duodenal gastrointestinal stromal tumor and periampullary carcinoma

The surgical and postoperative outcomes of these patients are shown in Table 3. None of the patients needed additional port insertion or conversion to laparotomy. Median operation time (222.5 (208.8–245.0) vs. 305.0 (256.0–337.0) min, *P* < 0.001) was shorter in SILPD + 1 than in CLPD. The estimated blood loss, blood-transfusion rate, exhaust time, time of drainage tube removal, and postoperative hospital stay were comparable between the two groups. Median postoperative VAS in day 1 (2.5 (2.0–3.0) vs. 3.0 (3.0–4.0), *P* < 0.001), day 2 (2.0 (1.75–2.0) vs. 2.0 (2.0–3.0)), *P* = 0.039) and day 3 (1.5 (1.0–2.0) vs. 2.0 (1.0–3.0), *P* = 0.001) were lower in SILPD + 1 group than in CLPD. The median cosmetic score (21.0 (19.0–23.5) vs. 17.0 (16.0–20.0), *P* = 0.026) was higher in SILPD + 1 than in CLPD one month after the surgery. The overall complications were comparable between the two groups. The patients in SILPD + 1 developed fewer grade B and C pancreatic fistulas (10.0% vs. 19.1%, *P* = 0.816), had lesser DGE (10 vs. 12.8%, *P* = 1.00), and fewer PPH (0 vs. 10.6%, *P* = 0.574) than those in the CLPD group. However, the difference was not significant.

**Table 3:** Intraoperative and postoperative outcomes of two groups

Variables	SILPD + 1 *n* = 10	CLPD *n* = 47	*P*
Operative time, median (IQR), min	222.5 (208.8–245.0)	305.0(256.0–337.0)	< 0.001
Venous resection, n (%)	2 (20.0)	0 (0)	0.028
Conversion, n (%)	0 (0)	0 (0)	1.000
Transfusion, n (%)	0 (0)	5 (10.6)	0.574
EBL, median (IQR), mL	75.0 (24.5–162.5)	100.0 (50.0–200.0)	0.101
POHS, median (IQR), d	14.0 (9.8–19.0)	14.0 (10.0–23.0)	0.562
Exhaust time, median (IQR), d	2.0 (2.0–3.0)	3.0 (2.0–4.0)	0.088
Time of drainage tube remove, median (IQR), d	9.0 (6.0–12.3)	9.0 (6.0–13.0)	0.975
Postoperative VAS, median (IQR), p			
Day1	2.5 (2.0–3.0)	3.0 (3.0–4.0)	< 0.001
Day 2	2.0 (1.75–2.0)	2.0 (2.0–3.0)	0.039
Day 3	1.5 (1.0–2.0)	2.0 (1.0–3.0)	0.001
Biochemical leakage, n, (%)	4 (40)	14 (29.8)	0.798
CR-POPF^a^, n (%)	1 (10.0)	9 (19.1)	0.816
Hemorrhage, n (%)	0 (0)	5 (10.6)	0.574
DGE, n (%)	1 (10.0)	6 (12.8)	1.000
Reoperation, n (%)	1 (10.0)	2 (4.3)	0.446
Chylous leakage, n (%)	1 (10.0)	2 (4.3)	0.446
Biliary fistula, n (%)	0 (0)	3 (6.4)	1.000
Abdominal infection, n (%)	0 (0)	1 (2.1)	1.000
Incision infection, n (%)	0 (0)	3 (6.4)	1.000
Clavien–Dindo classification	_	_	0.281
I-II, n (%)	6 (60.0)	17 (36.2)	_
≥ III, n (%)	1 (10.0)	3 (6.4)	_
90-Day Mortality, n (%)	0 (0)	1 (2.1)	1.000
Cosmetic score^b^, median (IQR), p	21.0 (19.0–23.5)	17.0 (16.0–20.0)	0.026

*SILPD* + *1* Single-incision plus one-port laparoscopic pancreaticoduodenectomy, *CLPD* Conventional laparoscopic pancreaticoduodenectomy, *SD* Standard deviation, *IQR* Interquartile range, *EBL* Estimated blood loss, *POHS* Postoperative hospital stay, *VAS* Visual analog scale, *CR-POPF* Clinical related postoperative pancreatic fistula, *DGE* Delayed gastric emptying

^a^CR-POPF: only includes grade B and grade C postoperative pancreatic fistula

^b^Cosmetic score: data were obtained by follow-up one month after the operation and 46 patients were included

## Discussion

SILS is an important branch of laparoscopic technology that has been performed in almost every field of surgery, such as general surgery, gynecology, urology, and thoracic [4, 5]. SILS offers potential benefits over conventional laparoscopic surgery, including a reduced risk of trocar-related complications, reduced postoperative pain, and improved convalescence and cosmetic results [17–19]. LPD is a more technical challenge, and the first research on SILPD was reported [6] in 2022. The main reason for the slow development of SILPD may be the technical challenges, conflicts of surgical devices, and the lack of triangulation and inline viewing [17–19].

Between March 2013 and December 2019, three pancreatic ductal adenocarcinoma patients were reported with longer operative time (481.7 min) and higher blood loss (800 mL) [6]. For highly selected patients, we began performing SILPD in May 2020 and finished 13 SILPDs. The median operative time was 340 (310–356) min, and the median EBL was 50 (50–125) mL (data not shown in this paper). We also found some other benefits like low pneumoperitoneal pressure and reducing the participation of assistants [7]. Compared with our 550 CLPDs in a previous study, SILPD had longer median operation time (340 (310–356) vs. 323.5 (250 –420) min) but lower median EBL (50 (50–125) vs. 200 (120–300) mL) [2]. We used only one scope port and two operation ports in SILPD, so the number of instruments was limited and patients should be highly selected [7]. In particular, the assistant cannot use the suction device to remove the blood and surgical smoke from time to time, which impaired the clarity of vision and required very careful dissociation to avoid bleeding in SILPD. Herein, the retraction devices made with bulldog forceps were extremely important [7]. We clamped the organs using the bulldog forceps and pulled the thread at the end of the forceps out of the abdominal wall to assist in time-consuming exposure. To decrease these difficulties, we have added one operating port for device manipulation, which no one had reported before. Through this port, the abovementioned technical challenges can be better resolved while preserving the minimally invasive benefits of SILPD. We can also use this port for drainage, which is very hard in SILPD. Thus, SILS + 1 surgery provides a more feasible option for LPD.

We also found the that operation time (222.5 (208.8–245.0) min) of SILPD + 1 decreased significantly, and it was even faster than that of the CLPD group (305.0 (256.0–337.0) min in this research and (323.5 (250–420) min) in a previous study) [2]. The main reasons were including highly selected patients and fewer assistant participants in SILDP + 1, which can reduce the influence of the assistants’ experience on operation time in CLPD. It was similar to the result in laparoscopic distal gastrectomy, and this difference was also attributed to using extracorporeal gastrojejunostomy for SILPD + 1 and intracorporeal gastrojejunostomy for CLPD [20]. However, SILPD + 1 resulted in a certain decrease in interference between instruments and scope compared with SILPD. We even finished two LPDs combined with vascular resection and reconstruction in SILPD + 1. No statistically significant differences were observed in postoperative complications. SILPD + 1 further showed lower postoperative pain and higher cosmetic results than the CLPD group. This finding was consistent with that of Liu [21] for rectosigmoid cancer. Therefore, the safety and effectiveness of this surgical method are supported.

Reduced‐port robotic pancreaticoduodenectomy has been reported by Cho‐Han Chiang [22]. They found that reduced-port RPD is associated with less blood loss but a longer operative time than OPD. Meanwhile, no studies have compared reduced‐port a robot to perform pancreaticoduodenectomy and SILPD or SILPD + 1. In theory, using a robotic to do single-port surgery has more advantages such as fewer assistants and less instrument interference. The limitations of the single-port surgical robot system included not being as popular as laparoscopy and the high cost in our country. Very few hospitals have this system.

The major limitation of this study was its retrospective nature with small sample size. As such, the treatment strategy was not based on random assignment, and the patients were highly selected. Another limitation was that data were obtained from a center with a high volume of patients, which may have influenced the results. Accordingly, we suggest conducting a multicenter prospective randomized controlled study in the future.

## Conclusion

In a high-volume LPD center, SILPD + 1 is safe and feasible for well-selected patients without increasing the operation time and complications. It even has the advantages of reduced postoperative pain and improved cosmetic results.

### Supplementary Information

**Additional file 1.**

## Data Availability

The datasets used and/or analysed during the current study are available from the corresponding author upon reasonable request.
